# Tiny Machine Learning Implementation for Guided Wave-Based Damage Localization

**DOI:** 10.3390/s25020578

**Published:** 2025-01-20

**Authors:** Jannik Henkmann, Vittorio Memmolo, Jochen Moll

**Affiliations:** 1 AG Terahertz-Photonik Physikalisches Institut, Johann Wolfgang Goethe-Universität, Max-von-Laue-Strasse 1, 60438 Frankfurt am Main, Germany; 2 Department of Industrial Engineering, Universitá degli Studi di Napoli ‘Federico II’, Via Claudio 21, 80125 Naples, Italy; vittorio.memmolo@unina.it; 3 Department of Mechanical Engineering, University of Siegen, Paul-Bonatz-Straße 9-11, 57076 Siegen, Germany

**Keywords:** edge AI, machine learning, ultrasonic guided waves, structural health monitoring, tiny device

## Abstract

This work leverages ultrasonic guided waves (UGWs) to detect and localize damage in structures using lightweight Artificial Intelligence (AI) models. It investigates the use of machine learning (ML) to train the effects of the damage on UGWs to the model. To reduce the number of trainable parameters, a physical signal processing approach is applied to the raw data before passing the data to the model. Starting from current state of the art in algorithms used for damage detection and localization, an AI-based technique is developed and validated on an experimental benchmark dataset before tiny ML implementation on a low-cost development board. A discussion of the need for a balance between the reduction in computational resources and increasing the precision of the models is also reported. It is shown that by extracting simple features of the signal, the models required to predict the damage locations can be significantly reduced in size while still having high accuracies of over 90%. In addition, it is possible to use these predictions to construct a fairly accurate heat map indicating the likely damage locations. Finally, a convenient edge/cloud visualization of the results can be achieved by simplifying the heat map.

## 1. Introduction

Structural integrity is crucial in modern as well as aging assets. Small damage to primary structures that goes unnoticed can lead to devastating effects down the line, such as breaking under sudden stress or fatigue before the expected end of life. For this reason, it is paramount to detect and to localize said damage to circumvent these types of problems. Structural Health Monitoring (SHM) is a fundamental asset for many industries and, as such, a key research topic. SHM aims at deriving the characteristics of mechanical structures and components by measuring signals from distributed transducers in order to identify any critical anomaly [1]. The characteristics derived from a SHM system can be categorized into different levels and classes based on the monitored information about the condition of the structure [2]:

- Binary damage indicator (classifying if there is a damage or defect);
- Position (localization, in addition to classification);
- Multi-class damage or defect (to distinguish different damage classes, usually connected to size assessment);
- Maintenance level (priority and time prediction);
- Lifetime and fatigue prediction (usability time prediction).

Classifying and localizing damage are the key predictions to warn of anomalies before investigating in detail the possible failure. To this end, it is crucial to retrieve useful information employing damage sensitive features gathered from the structure. These features can be extracted using different measuring technologies, e.g., measuring strains by strain gauges [3,4], acoustic emission (AE) signatures by piezoelectric transducer or fiber optic sensors [5,6], electromechanical impedance signature of the integrated transducers [7], and ultrasonic guided waves traveling within the media [8].

Among them, UGW-based approaches are quite promising and have been increasingly used in both research and industry. The UGW propagation can be exploited for AE algorithms or in pitch–catch measurements, actuating to and sensing from piezoelectric transducers. In general UGWs can investigate a large area using a limited number of sensors [9,10]. In addition, they are very sensitive to early damage and tunable to the specific application by setting the proper frequency and wave mode. In particular, monitoring wave propagation characteristics (e.g., time of flight, energy, and attenuation) can return damage information.

Most recent SHM methods are based on machine learning (ML) algorithms and neural networks (NN) [11,12,13]. Despite most of the available literature being focused on detecting a damage (binary prediction), some ML applications are specifically dealing with UGWs with the aim of localizing damage. Indeed, Deep Neural Networks (DNNs) can be used to assist in locating very small damage [14]. Many approaches combine experiments with simulation in order to increase the availability of data. Also, to reduce the number of measurements needed, a possible solution is represented by unsupervised machine learning methods [15]. However, DNNs are generally known to be very data-intensive ML algorithms that need a lot of training data to calculate the trainable parameters. To reduce the number of parameters, instead of training the NN on the entire signal, a filtered signal can be processed to have certain features extracted that can be used to interpret the state of the signal and its structure [16]. By comparing the signal to a reference baseline before feature extraction, the environmental influence can be reduced to a minimum, especially when a temperature compensation algorithm is adopted, and the damage can be identified by the signal changes. These extracted features remain similar when no actual modification occurs, and change when the structure is damaged. In principle, it is possible to define a function that takes all the features as an input and returns a probability of the structure being damaged. In practice, this requires uncountable experimental efforts, unless characterizing the system for a simple and specific case [17]. Here is where NNs come into play, as they can be used to find this underlying function by training the model over experimental data. However, even though the NN is able to find such a function, this is often hidden in the weights and the structure of the model, and therefore it is hard to reconstruct and interpret. This is the main reason why explainable approaches have recently been generally preferred [18].

In addition, decentralized SHM architectures are necessary to deal with a number of transducers disseminated in different locations. This requires using every transducer as an SHM node with computational capacity. This is the reason why it is worth looking at the tiny machine learning implementation of SHM on low-power devices with limited computing capabilities [19,20]. Despite the promising impact, ML on tiny devices is a new field with a limited amount of available research, especially in the context of SHM and UGW [21]. In addition, the implementation requires a keen deployment of the model. A commonly used method is to build and evaluate the ML model in Python with the help of the Tensorflow module and export the model with the Tensorflow Lite module to C++ to export the model and load it onto the tiny device with the TensorFlow Lite for Microcontroller framework [22,23,24]. This work tackles three overarching problems: (i) signal processing of the raw UGW signal, (ii) training and evaluating a NN model, and (iii) implementing the model on a tiny device. Naturally, these steps should not be looked at in complete isolation since they can influence each other (e.g., the number of extracted features influences the model size and structure, which in turn influence the computational load on the tiny device).

**Figure 1:**
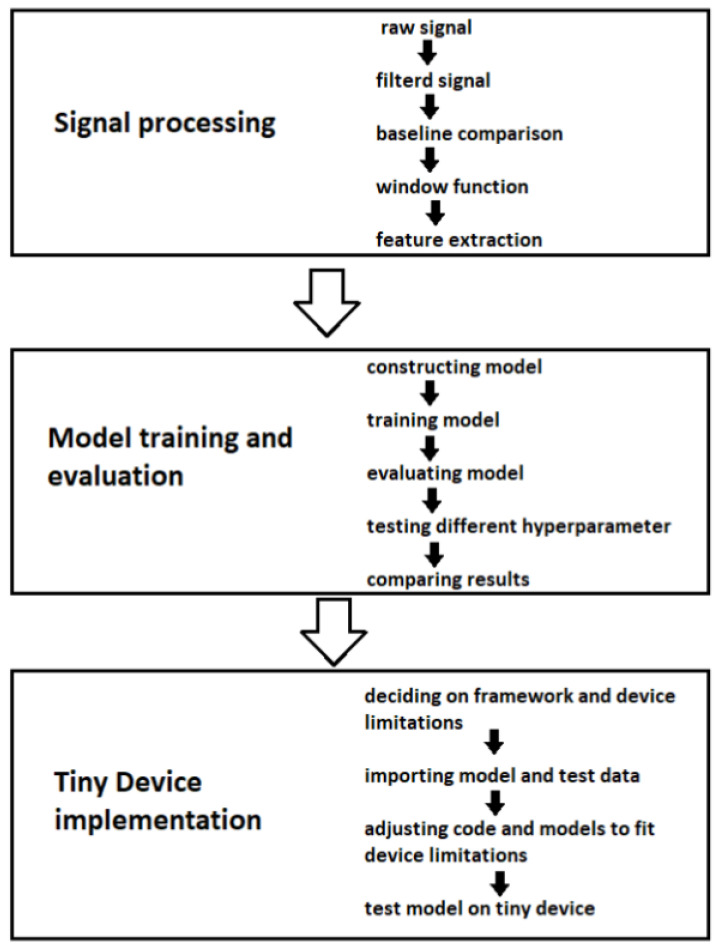
This figure shows the three themes with their corresponding steps performed in the work.

In this context, the paper proposes a stepwise approach to implement and deploy on the edge an ad hoc explainable data-driven NN model for damage detection and localization as shown in Figure 1. To achieve this goal, the publicly available dataset *Open Guided Waves* is exploited to obtain the UGW measurements necessary to build the ANN model up [25]. Then, this model is deployed on the edge to obtain a real-time low-computation diagnosis for the first time.

The remaining of the paper is organized as follows. The overall concept is first presented in the materials and methods section, before describing the dataset, the signal processing techniques, the NN model, the localization procedure and the edge implementation. Then, the results are shown to demonstrate the feasibility of the approach. Finally, the discussion and concluding remarks are reported.

## 2. Materials and Methods

This section aims to collect the methodological framework conceived. The overall concept is briefly presented before describing the dataset. Then, the signal processing techniques are detailed, and the NN model is described before explaining the rationale behind the localization procedure. Finally, the edge implementation approach is outlined.

### 2.1. Overall Concept

**Figure 2:**
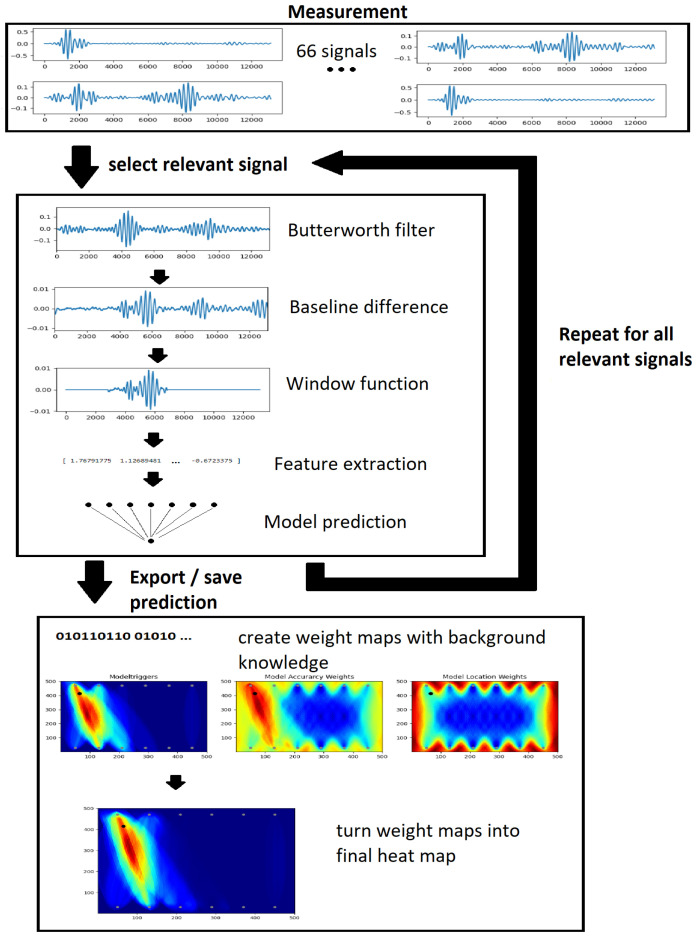
Schematization and visualization of data processing steps carried out on the measurement dataset from raw signals until the final prediction.

Ultimately, the goal is to take a UGW measurement dataset collected from a distributed sensor array and process it using neural networks to predict the sensor paths affected by damage and then use structural information from the system to create a heat map showing the most likely damage locations. The model predictions themselves are in binary format, but with the use of the structural knowledge of the plate and the transducers, the binary code can be turned into a heat map. The entire data processing flow can be seen in the flow chart in Figure 2. This shows the steps of the data processing that are carried out with the measurement until the final prediction. It starts with the measurement, which consists of 66 signals, from which a relevant signal subset is selected. All signals are then processed with a filter, and then the difference to the baseline is measured. The window function cuts out the pitched signal in the measurement, which is then passed on to the feature extraction. The model uses the extracted features to make a prediction for the signal. This is performed for all relevant signals. The results of the prediction can then be converted into weight maps using the structural knowledge of the covered plane and the models. These weight maps can then be used to calculate the final heat map.

### 2.2. Dataset

This work uses the Open Guided Wave (OGW) #1-Dataset [25], which is a freely available online dataset that consists of UGW measurements of 12 transducers on a 0.5 m × 0.5 m carbon fiber-reinforced polymer plate. One aluminum disk, simulating damage on the plate, has a diameter of 10 mm and a height of 2.35 mm, resulting in a weight of 0.5 g. These aluminum disks cause scattering with UGWs [26] that are sent out by the transducers as acoustic waves with frequencies ranging from 40 kHz to 260 kHz in steps of 20 kHz. This signal change is considered a “damage” in this paper.

Arbitrary wave forms are generated by a Handyscope HS5 (TiePie Engineering) while signals are recorded by an analog-to-digital converter (14-bit resolution). Next, a broadband amplifier PD200 (Piezo Drive) amplifies the excitation wave forms and forwards the signals to a custom multiplexer. This device allows measuring all actuator–receiver combinations by time-division multiplexing and storing the signals in the HDF5 file format. The time duration of each ultrasound measurement is approximately 1.31 ms.

To keep the environmental influences to a minimum, the measurements are performed in a climate chamber at 23 °C and 50% relative humidity. Two temperature sensors are installed on the top left and bottom left to measure the surface temperature of the samples.

**Figure 3:**
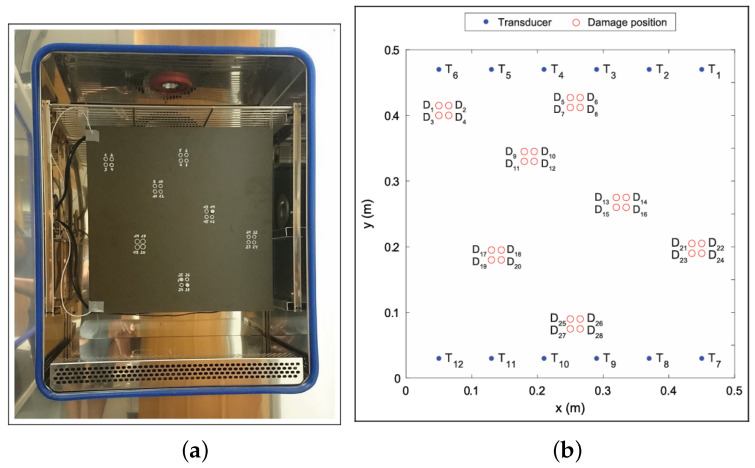
This figure shows on the left (**a**) the SHM plate inside of the climate chamber, with the damage locations marked with white circles, and on the right (**b**) the graph showing the named damage locations in the coordinate system.

Figure 3 shows the experimental setup within the climate chamber along with the scheme of the transducer deployment, consisting of 12 sensors in two rows of six transducers. The deployment comes from discussions with aircraft original equipment manufacturers for the preparation of the initial dataset suitable for aerospace application. The coordinates of these transducers are reported in Table 1.

Measurements are made for 28 different damage locations and 60 baseline measurements (20 on each day of tests). For each possible transducer pair (TP), one signal is measured, which leads to 66 signals. This is performed for the excitation frequencies from 40 kHz to 260 kHz by increasing the frequency by 20 kHz at each step, but for the final model set developed, only the 40 kHz measurements are used [25].

### 2.3. Signal Processing (SP) and Feature Extraction (FE)

**Figure 4:**
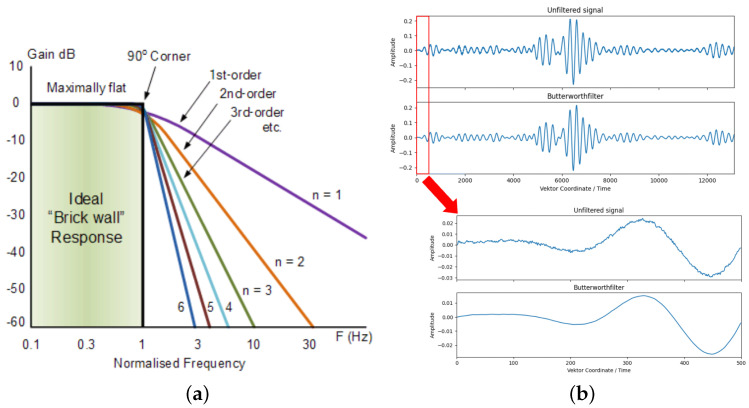
This figure shows on the left (**a**) the gain response of a low-pass BWF for a normalized frequency (target frequency = 1). On the right is (**b**) the raw signal is compared to the filtered signal using a pass-band BWF of the first order with a target frequency of 40 kHz.

The dataset consists of the raw measurement data that are further processed with the Butterworth filter (BWF). The BWF is designed to have a frequency response that is as flat as possible in the pass band (around the signal frequency). This is why it is also referred to as the “maximally flat magnitude filter”. The maximally flat response is achieved with no pass band ripple until the cut-off frequency at −3 dB and a roll-off of −20 dB in the stop band (per decimal power). A major disadvantage of the BWF is that it achieves this pass band flatness at the expense of a wide transition band as the filter changes from the pass band to the stop band. This can be corrected by using higher orders of the BWF. Figure 4 shows the influence of the BWF to a signal. Figure 4a displays how the suppression of non-target frequencies becomes stronger with higher orders. However, higher orders also introduce an increasing time lag in the signal, suggesting to keep the order as low as possible when the frequency filtering is enough. Figure 4b shows that the first-order BWF applied to one of the signals reduces the noise strongly, which is the reason why higher orders are not deemed necessary.

After applying the BWF to the signals, the master baselines are constructed, taking the mean of the baseline measurements. Since the experiments are carried out in a climate chamber with constant environmental settings, all 60 baselines are used to create one master baseline that is used for all damage types. This helps in making the model more robust towards smaller influences. The same action is performed for each considered channel (transducer pair) and frequency. In the end, there is a total of 66 master baselines for each frequency (the final model set uses only the 40 kHz data).

As a final step before the FE, a window function cuts out the pitched signal from the measurement, with the help of the correlation function, which finds the position of the highest correlation between the pitched signal and the received signal. The correlation *z* between 2 arrays x,y is calculated with the formula

(1) $$z\left(k\right)==x*y\left(k-N+1\right)=\sum_{i}^{||x||-1}x_{i}y_{i-k+N-1}^{*}$$

k=[0,||x||+||y||−2]; ||x|| length of x, N = max(||x||,||y||), $y_{m}$ is 0 when m>||y|| [27].

**Figure 5:**
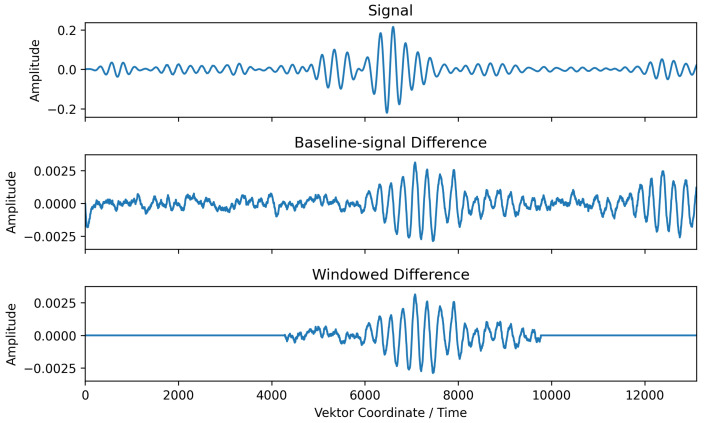
Effect of window function applied to the difference between the measured signal and the corresponding master baseline.

The window is then set to 1500 values (which corresponds to 0.15 ms) before this position (*k* corresponding to $z_{max}$) to make sure to include the whole signal in the window, and to 5500 values (0.55 ms) after this position to include some scattered waves, too. This is performed because it can be assumed that the relevant information about the damage will be on the pitched signal or at least in its direct vicinity. The window is then applied on the difference of the received signal to the master baseline. Figure 5 shows the effect of the window function applied to the difference between the measured signal and the corresponding master baseline. As a consequence, the rest of the vector is obtained padding zero elements. When applied in code, only the non-zero part is used for further processing.

The extracted features are the Root Mean Squared Deviation (RMSD), variance, standard variation, the mean value of the window (to look at the deviation from zero), the global maximum (position and value) and the position of the pitched signal in the received signal (i.e., time of flight).

The RMSD compares two signals (or vectors) and qualifies their difference as a singular value. This can be used to get an idea of the divergence of the signal (defect installed) to the baseline (without defect). The mathematical expression is as follows:

(2) $$RMSD=\sqrt{\frac{\sum_{i=0}^{n}{\left(S_{b}\left(i\right)-S_{l}\left(i\right)\right)}^{2}}{\sum_{i=0}^{n}S_{b}{\left(i\right)}^{2}}}$$

Here, $S_{b}\left(i\right)$ is the i-th element of the baseline vector, and $S_{L}\left(i\right)$ is the i-th element of the signal vector. n is the overall length of the signal vectors, or the length of the considered part (for the RMSD value) of the signal [28].

The positional features are normalized towards the length of the signal, and all other values are scaled to be in the range of 0 to 1 because neural networks have an easier time of calculating the weights when all inputs are in a similar range.

### 2.4. Neural Networks

The ML algorithm used consists of 40 one- to two-layer deep categorization NNs. The algorithm is developed using the Python library Tensorflow which is then exported to a C++ Framework with the Tensorflow light library. The NNs use a ReLU activation function and the Binary Cross Entropy as a loss function with the Adam algorithm optimizer.

**Figure 6:**
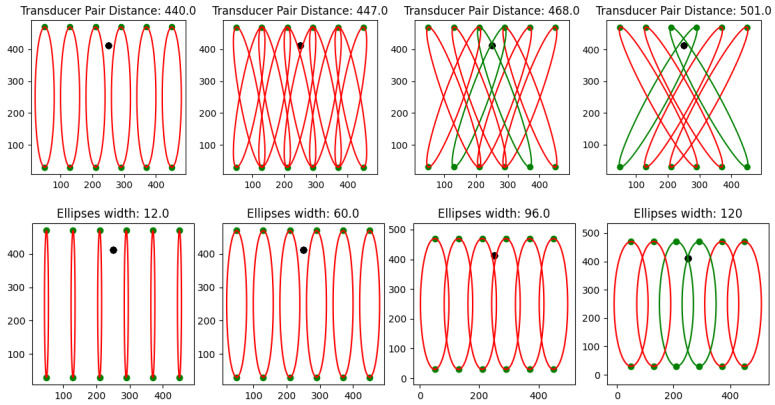
(**Top**) Ellipses for the different TP-distances (in [mm]) with a width scale of 60 mm. (**Bottom**) Ellipses for different width scales (in [mm]) for a constant distance of 440 mm. Green ellipses includes example damage.

One NN takes the extracted features for one TP and predicts whether there is damage in between the TP or not. Different models are trained for different distances between the TP and for different sized trigger areas. An ellipse is drawn using the two transducers as edge points, and a scaling factor is used to define the width of the ellipse. A binary classification is consequently adopted and returns 1 when the damage is predicted inside the defined ellipse, while it returns 0 otherwise. This is performed for 4 different distances between transducers (440 mm, 447 mm, 468 mm and 501 mm) and 10 different widths of ellipses (12 mm, 24 mm,..., 120 mm), which leads to a total of 40 models. This specific width is chosen because it corresponds to 1.5 times the distance of the neighboring transducer and accomplishes good coverage of the entire plate. Figure 6 shows different constructed ellipses/models covering the plate area. At the top, the ellipses for the different TP-distances are shown with a width scale of 60 mm. At the bottom, the ellipses for different width scales and a constant distance of (l = 440 mm) are shown. Green ellipses have the example damage ($D_{7}$) inside and will return a 1, while the red ellipses have the example damage not inside and return a 0 for the model. Each of these pictures represents one (different) model that is used multiple times, namely, once for each of the ellipses in the corresponding picture.

### 2.5. Heat Map Construction

By layering the results of the different models, a heat map can be constructed. However, it is worth noting that different spots on the plane are covered by a varying amount of models. That is why some model results have a stronger influence on the heat map than others. To overcome this inhomogeneity, an additional algorithm is developed to weight the different model results and reduce the strong influence of single models.

**Figure 7:**
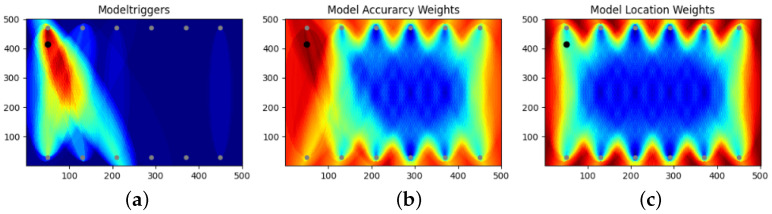
Comparison of different weight maps: (**a**) number of triggers on the CP (trigger map), (**b**) triggers and model counters weighted with the accuracy of the models (probability map), and (**c**) inverse model counter map (blue has many models covering the CP, while red means few or no models covering the CP).

The typical result is reported in Figure 7. The algorithm constructing the heat map that operates threefold calculations has three main steps: (i) counting the number of models that look at each coordinate point (CP) or spot (depending on resolution), (ii) counting how many of these models return a damage prediction for this CP, and (iii) weighting the accuracy of the model. This way, two heat maps are constructed from the first two steps: the model counter map (which is the same for each damage or measurement), and the model trigger map. These two maps are further combined into a probability map, which also uses the approximate accuracy of the model to consider false triggers.

By combining these three maps, the final heat map is constructed as follows:

(3) $$P\left(x_{i},y_{i}\right)=\left(0.95^{T(x_{i},y_{i})}*0.05^{M\left(x_{i},y_{i}\right)-T\left(x_{i},y_{i}\right)}\right)$$

where we have the following:

P($x_{i},y_{i}$) is the first probability map weight for the CP at ($x_{i},y_{i}$).
T($x_{i},y_{i}$) is the amount of triggered models for the CP at ($x_{i},y_{i}$).
M($x_{i},y_{i}$) is the amount of models covering the CP at ($x_{i},y_{i}$).
0.95 is the average accuracy of the models used.
0.05 is the average probability of a false trigger.

For the final probability map, the map is further scaled as follows:

(4) $$P_{2}\left(x_{i},y_{i}\right)=\frac{log(P\left(x_{i},y_{i}\right)-log\left(P_{min}\right)}{\left|log(P_{min}+1.1)\right|}$$

The weight maps are combined with the formula

(5) $$F\left(x_{i},y_{i}\right)=\frac{T(x_{i},y_{i})}{log(M\left(x_{i},y_{i}\right)+1.1)}\cdot P_{2}\left(x_{i},y_{i}\right)$$

To give a better explanation of the weighting procedure, it is worth noting that Equation (3) is meant to provide the probabilistic weights including the possibility of models making wrong predictions. To this end, the average accuracy (equal to 0.95) of the models is weighted by all triggers, while the probability for a false prediction (equal to 0.05) is weighted by the difference between the number of models that cover the given coordinate point and the ones triggered. This is the most simple yet effective attempt to include the model accuracies to the weights. Equation (4) scales the results of Equation (3) to get rid of some artifacts and manage the influence of Equation (4) in Equation (5). This scaling (the logarithm) is adjustable and influences the localization and production of artifacts and can be fine-tuned further. In Equation (5), the relation of the actual number of triggered models and models covering the coordinate point is merged with these probabilistic weights. The logarithm is used for scaling reasons, while the + 1.1 constant factor is introduced to prevent artifacts from dividing through 0.

It is worth noting that this weighing, together with the model training, still leaves room for optimization but delivers satisfying results for the singular damage measurements. The actual accuracy of the model can be used to increase the localization accuracy, but it is not implemented to achieve a good balance between effectiveness and efficiency. Indeed, this optimization needs additional information to be saved, exported to the tiny device format, and loaded into its memory. Furthermore, comparisons of different parameter results would not be possible due to the loose consistency of the heat maps.

**Figure 8:**
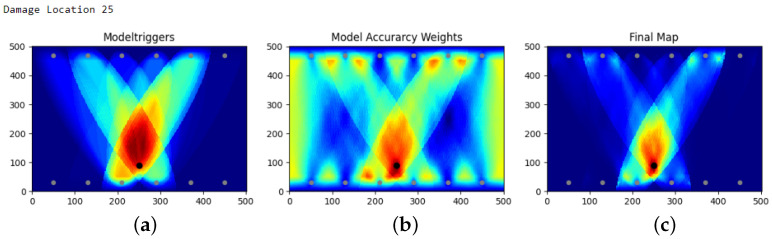
Heat map composition including (**a**) trigger map. T($x_{i},y_{i}$), which just counts the number of triggered models, (**b**) probability map, $P_{2}$($x_{i},y_{i}$), which is calculated via (4), and (**c**) the final probability map, F($x_{i},y_{i}$).

In addition, the color depth of the heat map is obtained via the Python module matplotlib, which uses an adaptive scale for the data (Figure 8). This approach has the disadvantage that the same color (e.g., red) in one graph represents a different value than the same color (e.g., red) in another plot. The maximum values of the heat maps can vary between 10,000 and 400,000; the reason for the big variance is likely the model coverage problem of the different CPs on the map (CPs have different amounts of models looking at them). This could be fixed by normalizing the data, with the direct consequence that the information needed to identify whether or not the plate is damaged gets lost, or at least returns non-reliable prediction. This is because relatively low maximum values (<5000) can be used to identify an undamaged plate with high accuracy. This is tested with baseline measurements whose maximum values vary between 0 and 7000, with an exception (which can be seen as a false damage prediction) of around 18,000.

### 2.6. Tiny Device Implementation

The tiny device used is the ESP32-S3-DevKitC-1-N8 with 320 kB of RAM and 8 MB of ROM from ESPRESSIF. The edge device has two USB ports, which are used to configure and send data to the device. It can also be accessed via Bluetooth and WiFi, but these features are not used or tested. A legend explaining the different parts in more detail can be found in the official documentation [29].

**Figure 9:**
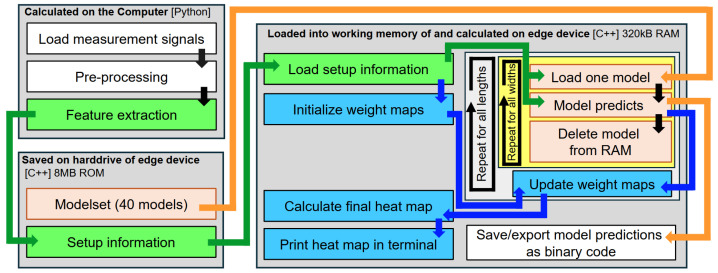
Flow chart picturing the different actions executed on the chip and the preparations performed in Python.

To program the chip, the relevant drivers are downloaded from its official website [29] and the programming is performed in the Visual Studios (VS) application https://platformio.org/ Platform IO. For this purpose, a GitHub-repository [30] is copied and used as a base reference. Figure 9 shows a flow chart picturing the different steps completed on the chip and the preparations performed in Python. The three gray boxes represent the different kinds of computational resources used. The feature extraction is performed not on the tiny device but on the computer using Python (upper left gray box). The extracted features are saved together with the setup information and the entire final model set (which is trained and optimized in Python on the PC) on the drive of the edge device (lower left gray box). The setup information includes the transducer positions, the ellipses function, and information about what features belong to what TP. The right gray box represents the RAM of the tiny device. It shows what has to be loaded into the working memory and is calculated directly on the edge device. The colors represent the different kinds of information. Orange follows the models, green follows the features (and TP-information), and blue follows the information for the heat map construction. The white boxes are the input and output of the total system, and the black arrows represent a straightforward calculation or process step. The two smaller boxes (light gray and yellow) represent two loops that load one model after the other, iterating over the width and length of the ellipses that correspond to the models.

It is worth noting that memory allocation is a key step to properly deploy the models on the edge. Having 8 MB ROM, all models of the model set (in C model format), together with the model input (the extracted features) and the structural information of the SHM plate (TPs and TP positions), can be saved on the chip properly. However, the feature extraction requires all measured signals together with its master baselines to be saved on the ROM of the chip, which is not feasible. For this reason, the extracted features calculated in Python are exported to the chip directly. This issue can be circumvented in real-time application by streaming one signal at a time to the chip, extracting and saving the features before deleting the signal and streaming data again.

It is worth noting that the blue boxes in Figure 9 are not needed for the model predictions and calculations. Even the setup information can be reduced to the extracted features (and the TP-source information), which could also be handled by a continuous connection to the measurement system. This information is only needed if the heat map is also calculated on the edge device. However, this step can be executed on the cloud or after exporting the results as a short binary code to the end user as shown in the results section.

## 3. Results

This section shows the exemplary results demonstrating the feasibility of this approach and the preliminary execution on the edge. First, the model training is shown, detailing the model behavior having in mind to deploy a pre-trained model on the edge. Then, the classification results are reported, having a look at the localization capability of the trained models. Finally, the edge deployment and light implementation of the localization map are discussed along with the results streaming possibilities.

### 3.1. Model Training

In training, the 40 different models are trained in one big loop. In this loop, the training of a single model is further divided into training sets (e.g., 10 sets of 1000 training epochs). After each set, the code checks if the model became stuck in training and, if so, introduces smaller changes in an attempt to get the model weights out of the local minimum. These smaller changes include a resetting of the weights, changing the learning rate, and introducing a drop-out layer. Even with that, some models still become stuck in a local minimum. The main disadvantage is that singular models cannot be further optimized to reach their maximal potential, but in turn, this loop makes it easier to compare the influences of different model inputs (different features and/or signal processing steps) and model structures (different loss functions and/or layers/model sizes). Another criticality is related to the relatively small amount of training data for a relatively large number of trainable parameters. One model has 64 trainable parameters, which means having 2560 parameters to be trained in total with only 28 measurements. As a consequence, the training dataset is artificially increased by adding Gaussian noise to the raw signals, which also should make the models more robust towards smaller changes (e.g., environmental influences or electronic noise) in the signals. This is performed by calculating the standard deviations from the differences between the master baseline and the baselines and then taking the mean of the standard deviations to create an artificial time history with a random Gaussian noise function [31]. This noise vector is then added to the ”raw” damage signal before the signal processing. With this noise, the dataset is increased by three to five times.

**Figure 10:**
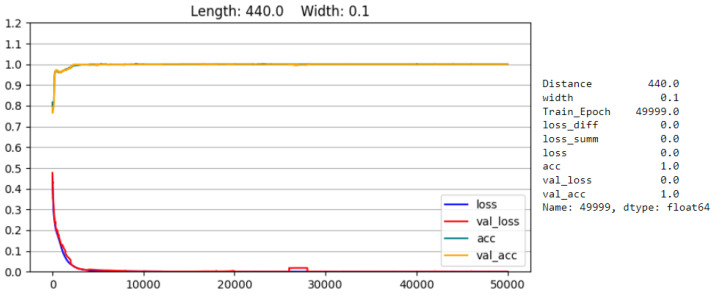

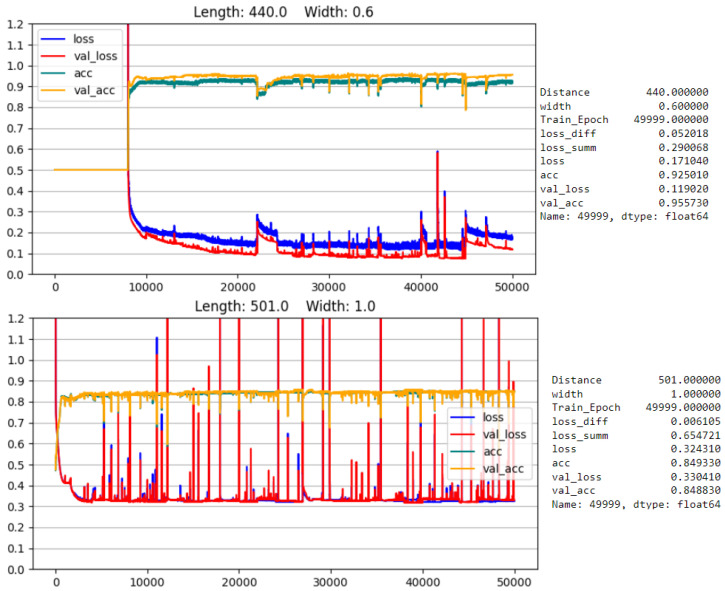
This figure shows a training overview for three models of the final model set. The x-axis shows the training epoch, and the y-axis is the numerical value of the loss and acc (from 0 to 1). At the top is an example of one of the most accurate models; in the middle is one of the more average models; and at the bottom is the model with the lowest accuracy. The distance (length) is noted in [mm], and the width in the arbitrary units of 120 mm = 1.0 → 0.1 = 12 mm.

For each parameter set, one model set is trained. An example of singular model training results (for the final model set) can be seen in Figure 10. The training results consist of the results for the training data (which are called *loss* and acc in the graphs) and the results for the evaluation data (which are called *val_loss* and *val_acc* in the graphs). The loss represents the average result of the loss function on this dataset, while accuracy (acc) describes the average rate for correct predictions of the model. For the evaluation (or test) dataset, one damage of each of the squares (which arrange four damages together; see Figure 3b) is taken out of the training data to evaluate the models. The different damage scenarios taken out of the squares have alternating relative positions: upper right corner for the first damage batch, lower left corner for the second, lower right for the third, and so forth. The evaluation damage locations are [$D_{2}$, $D_{7}$, $D_{12}$, $D_{13}$, $D_{18}$, $D_{23}$, $D_{28}$]. Furthermore, to prevent models from learning arbitrary biases, the actual training data are a subset that has one half its training data return a “0” and the other a “1”.

**Figure 11:**
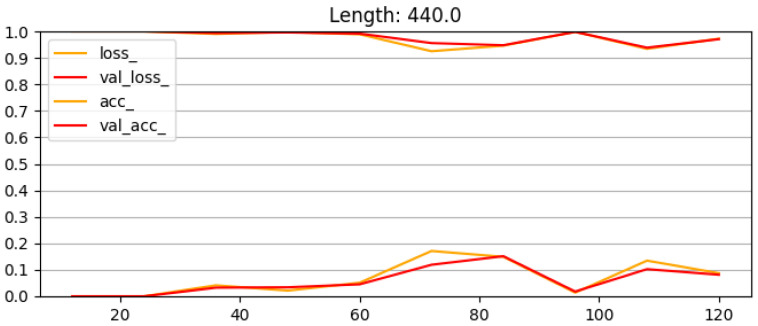

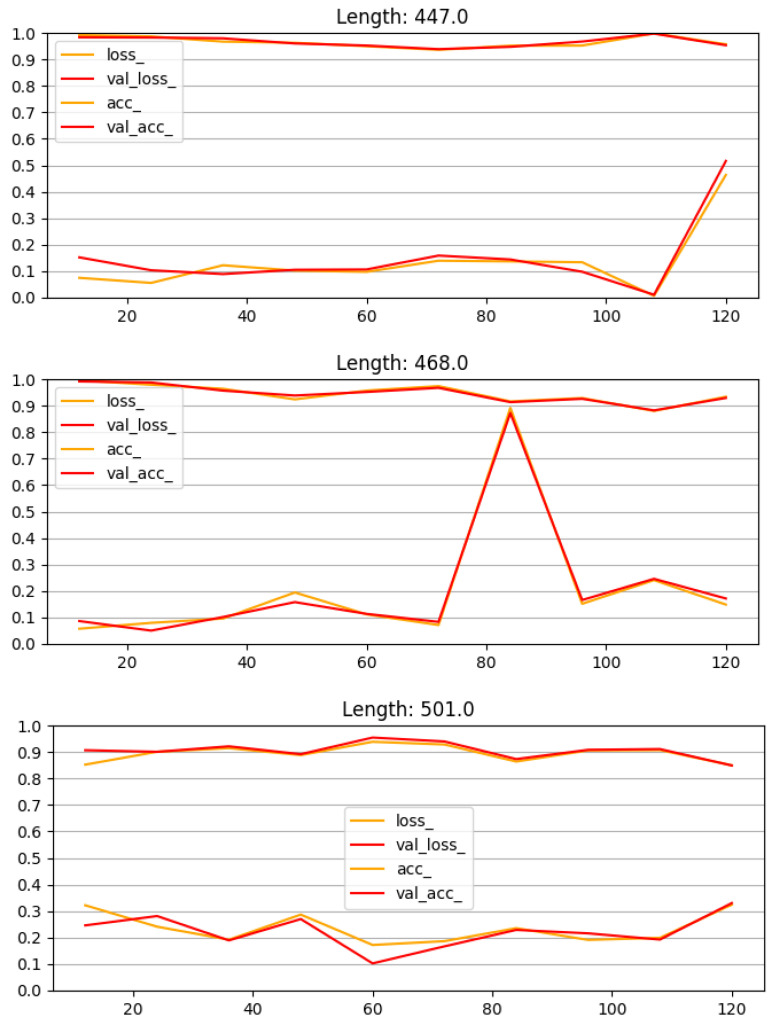
This figure shows an overview of the accuracies and losses for the final model set. With the different widths on the x-axis in [mm] and the accuracy (lines between 0.8 and 1) and loss (lines between 0.0 and 0.5 with one exception) on the y-axis, the orange lines represent the results for the training dataset, while red lines show the results of the evaluation (test) dataset.

Likewise, Figure 11 shows an overview of the accuracies and losses for the final model set at different lengths. The peaks in the losses are training cycles where the model becomes stuck in the training loop at higher losses. These ones could be retrained manually outside of the loop, but this action is not performed because they still have comparable accuracy to the other training results. It is worth noting that the training is not performed on the edge device and the models achieve an acceptable accuracy which means that they consistently reach over 90% accuracy.

### 3.2. Heat Maps

**Figure 12:**
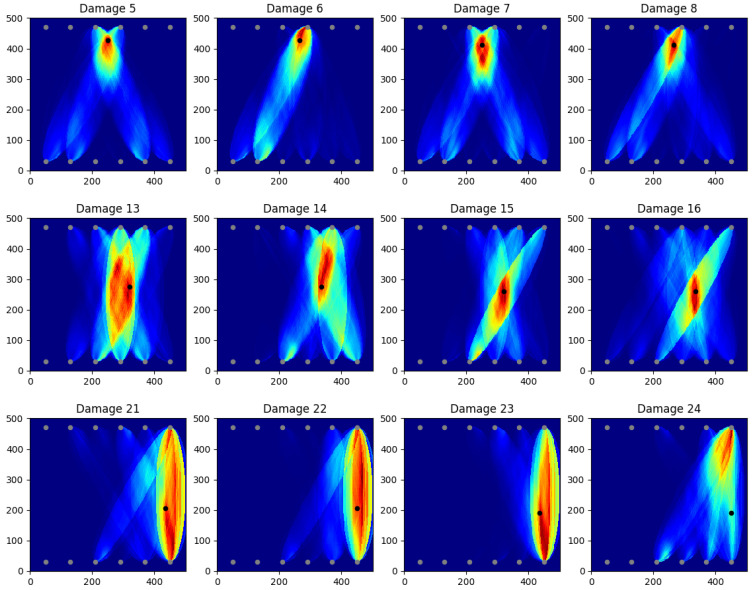
Model predictions turned into heat maps for 12 damage locations (black dots).

Some of the heat map results obtained with the aforementioned model are displayed in Figure 12. In particular, the first row shows the areas with the average best localization results (the borders close to the transducers); the second row shows the areas that have still quite good localization which can vary a bit (in the middle of the SHM-Plate); and the last row shows the area that has the worst localizations (at the edges without transducers). The last row also contains the only heat map which could be classified as a wrong prediction (damage 24), which is the only heat map where the actual damage is not in the red area (even though it is still in the highlighted area). However, taking a better look into this damage prediction, it is worth noting that this area of the plate is characterized by a poor coverage, making the localization challenging. However, this issue can be mitigated by having extended the coverage. Generally, it is worth noting that this approach has very high accuracy in terms of having the damage location in the highlighted area. Indeed, not a single heat map predicts the damage on a completely different side of the plate.

It is also worth pointing out that the heat maps only predict damage locations and do not classify if there is damage or not. This prediction is instead carried out by the NN models, looking into the damage occurring within the ellipse. In the damage classification approach, among the 60 baseline measurements and 28 damage location measurements, three would have been falsely identified as damaged/not damaged, returning a very high accuracy.

### 3.3. Tiny Device Results

**Figure 13:**
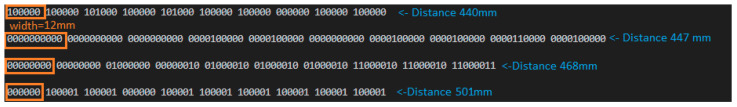
Visualization of model results in binary code form. One binary represents the prediction result from one model for one TP, one width, and one distance. One block (orange square) represents all TPs for one TP-distance and one width. Each line stands for all widths (from left to right, 12 mm width to 120 mm width in 12 mm steps) and for one TP-distance. (Done for Damage $D_{27}$).

**Figure 14:**
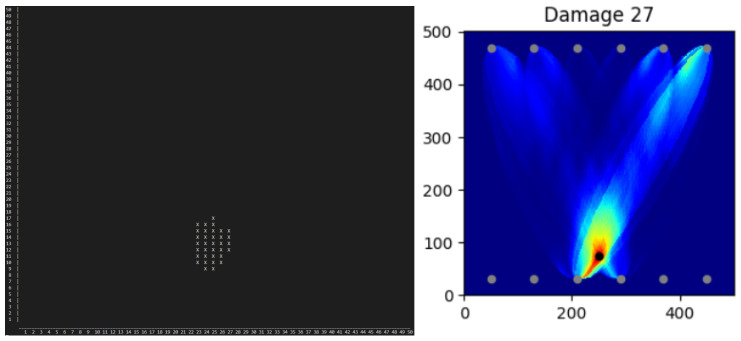
On the left are the binary results from Figure 13 turned into a simplified heat map on the tiny device, with the x- and y-axes in units of [cm]. X represents the predicted damage location. Comparison to the heat map made in Python for the corresponding damage location of 27 to the right.

Deploying the pre-trained models on the edge, the same calculations can be made directly on the chip to eventually export the results as binary code (see Figure 13 for $D_{27}$) or visualize a simplified heat map directly on the edge (see Figure 14 $D_{27}$).

The former one has the big advantage of being easily transferred to the cloud and/or to a workstation for further processing. Indeed, by introducing a wireless connection, the binary code can be streamed and passed into a reconstruction algorithm that decrypts the code, as it includes all the information needed to build the heat map up. Indeed, each binary in a block represents the prediction result from one model for one TP, one width, and one distance. One block (orange square) represents all TPs for one TP-distance and one width. Each line stands for all widths (from left to right, 12 mm width to 120 mm width in 12 mm steps) and for one TP-distance.

**Figure 15:**
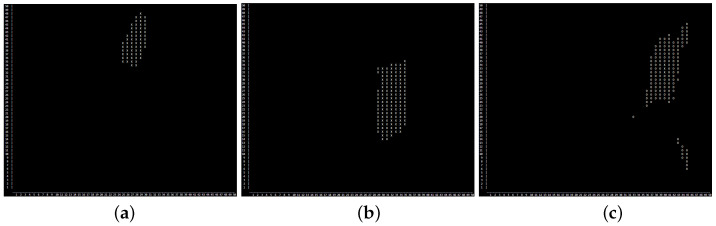
Simplified heat map for the 3 exemplary damage locations Damage 6 (**a**) (example for good localization), Damage 15 (**b**) (example for mediocre localization) and Damage 24 (**c**) (example for worst localization). The CPs with a value bigger than 0.9 (after normalization) are marked with an X and values between 0.5 and 0.9 with an O.

The latter one has the big advantage of visualizing coarsely yet clearly the location of damage in real-time on the chip and can be used as a first visualization option. A similar result is obtained in Figure 15, where the prediction of damage location is also normalized and the visualization is divided into two different ranges, having *x* for high probability and *o* for medium probability.

As a final remark, it is worth mentioning that the process runs smoothly on the edge and can be still expanded when needed. Indeed, the PlatformIO interface of the system shows 29% RAM and 22% flash usage during the initialization, leaving space for model increase when the code runs predictions with dynamic allocation (model removal after prediction).

### 3.4. Discussion

This work answers the question of whether it is possible to use machine learning algorithms on tiny devices to localize damage using ultrasonic guided waves. It is shown that damage localization can be realized with a limited amount of computational resources by training an appropriate set of small classification models (instead of a large localization model) to detect the presence of damage locally (between pairs of transducers) and then using their predictions to create a heat map with a reconstruction algorithm.

The models use extracted features from the transducer signals, while the algorithm estimates the location of the damage using a probabilistic distribution function. With this approach, a very high accuracy for the individual models as well as a good localization in the heat maps could be achieved. It should also be kept in mind that the experimental setup of the open-source dataset used is not optimized for this approach and can be improved, as most of the localization problems in the heat maps occur at the edges of the plane where the coverage by the transducers is limited. This problem could be improved by rearranging the transducers to improve coverage or by simply increasing the number of transducers at the edges. Furthermore, by reducing the sampling frequency and/or introducing a measuring system that includes automatic filtering and/or windowing, the size of the raw signal could be reduced, which in turn would reduce the resources needed for feature extraction.

Also, both the models and heat maps still show room for improvement and further optimization. Moreover, while the models reach high accuracy, the training results are inconsistent and sometimes show problems within the training loop. The natural solution would be to train each model in the model set individually and optimize the models that show problems during training. In addition, other shapes for the classification and features of the signals can still be explored.

The heat map construction is one of the areas that can be improved the most. Since the focus of this work is to develop a minimalist ML algorithm with acceptable accuracy, and the produced heat maps already show that the models can produce visible localization, further optimization is deemed unnecessary. Additionally, it can be seen that even in predictions where the heat map is a little off, the damage is still close to the predicted locations (Figure 12).

As for the tiny implementation, it is possible to partially replicate the system on a chip so that the testing and visualization are performed directly at the edge. Most of the resources are used for extracting the features of the signals, or, to be more precise, for signal processing and windowing, as the entire signal has to be loaded into the RAM. This can probably be avoided by adapting the code so that only parts of the signal are loaded to calculate the features step by step and store only those features on the edge device. Other ways to reduce the effort are the reduction in the sampling frequency or importing the extracted features directly to the tiny device after the signal has been pre-processed (e.g., by the measuring system). However, it is worth noting that the system is able to process the data, and the result can also be displayed in a simplified heat map before the diagnosis is sent in the form of binary code for further processing.

Finally, it is worth pointing out that the various model sets studied show a significant rate of variability in terms of the usage of different forms, signal processing/feature extraction steps, model parameters, and model set sizes. And this could indicate a high level of flexibility when applying this research to different setups or systems in which these models can be used once trained for these specific systems.

## 4. Concluding Remarks

This paper introduces an explainable neural network model applied to ultrasonic guided waves for damage localization where the damage presence is predicted locally (among a number of transducers couples), and then the retrieved information is globally exploited to localize the damage (through an imaging reconstruction). This approach allows to achieve good classification accuracy (always higher than 90%) while keeping the memory allocation very low, which makes this architecture well suited for edge deployment. The edge implementation shows the feasibility of a decentralized architecture with the prediction model running on the chip and displaying a simplified localization while streaming the information to the central unit for higher-resolution mapping.

## Figures and Tables

**Table 1:** Coordinates of the 12 transducers deployed over the plate.

Transducer	x-Coordinate [m]	y-Coordinate [m]
$T_{1}$	0.45	0.47
$T_{2}$	0.37	0.47
$T_{3}$	0.29	0.47
$T_{4}$	0.21	0.47
$T_{5}$	0.13	0.47
$T_{6}$	0.05	0.47
$T_{7}$	0.45	0.03
$T_{8}$	0.37	0.03
$T_{9}$	0.29	0.03
$T_{1}0$	0.21	0.03
$T_{1}1$	0.13	0.03
$T_{1}2$	0.05	0.03

## Data Availability

The training data used in this work are freely accessible at https://openguidedwaves.de/downloads/ as “OGW dataset #1” as well as papers describing the setup and measuring process.

## Abbreviations

The following abbreviations are used in this manuscript:

SHM	Structural Health Monitoring
UGW	Ultrasonic Guided Waves
OGW	Open Guided Waves
ML	Machine Learning
AE	Acoustic Emission
DL	Deep Learning
NN	Neural Network
RNN	Recurrent Neural Network
CNN	Convolution Neural Network
ToF	Time of Flight
RMSD	Root Mean Squared Deviation
ReLU	Rectified Linear Unit
BCE	Binary Cross-Entropy
MAE	Mean Absolute Error
AdaGrad	Adaptive Gradient Algorithm
RMSProp	Root Mean Square Propagation
PZT	Lead Zirconate Titanate
BWF	Butterworth Filter
TP	Transducer Pair
CP	Coordinate Point

SP	Signal Processing
FE	Feature Extraction
VS	Visual Studio
PIO	Platform IO
